# Anatomic Twist to a Straightforward Ablation

**Published:** 2013-03-07

**Authors:** Mandeep Singh Randhawa, Harris C Taylor, Robert D Mosteller

**Affiliations:** 1 Clinical Associate, Department of Hospital Medicine, Cleveland Clinic Foundation, Cleveland, Ohio; 2 Director Resident Research, Fairview Hospital - Cleveland Clinic, Clinical Professor of Medicine, Case Western Reserve University, School of Medicine, Cleveland, Ohio; 3 Cardiology/Electrophysiology, Heart and Vascular Institute, Cleveland Clinic, Cleveland, Ohio

**Keywords:** AV junction ablation, Inferior approach, IVC interruption, Azygos vein continuation, Superior vena cava, Heterotaxy syndrome, SRO sheath

## Abstract

Atrioventricular (AV) junction ablation for treatment of refractory atrial fibrillation is a well defined, standardized procedure and the simplest of commonly performed radiofrequency ablations in the field of cardiac electrophysiology. We report successful AV junction ablation using an inferior approach in a case of inferior vena cava interruption. Inability during the procedure to initially pass the ablation catheter into the right ventricle, combined with low amplitude electrograms, led to suspicion of an anatomic abnormality. This was determined to be a heterotaxy syndrome with inferior vena cava interruption and azygos continuation, draining in turn into the superior vena cava. Advancing Schwartz right 0 (SRO) sheath through the venous abnormality into the right atrium allowed adequate catheter stability to successfully induce complete AV block with radiofrequency energy.

## Case Summary

An 84 year old white female presented in March 2010 with dizziness, palpitations and shortness of breath. She was known to be hypertensive and diabetic, with a prior echocardiography showing evidence of moderate left ventricular hypertrophy, mild aortic and mitral stenosis and mild left atrial enlargement. No wall motion abnormalities consistent with significant ischemic heart disease were noted. She had previously been diagnosed with paroxysmal atrial fibrillation and tachycardia-bradycardia syndrome. After undergoing multiple cardioversions and failing antiarrhythmic drug therapy, the patient had settled into permanent atrial fibrillation by 2008. Acceptable rate control was challenging, and AV nodal blocking drugs produced occasional severe bradycardia and syncope, leading to placement of a Medtronic VVIR pacemaker. Despite subsequent aggressive AV nodal blockade with metoprolol 100mg twice daily, digoxin 0.125mg daily, and verapamil 240mg daily, she frequently experienced rapid ventricular responses to her atrial fibrillation, with associated symptoms of palpitations, dizziness, and diastolic heart failure. Additional AV nodal blocker therapy was relatively contraindicated due to chronic systolic blood pressures of approximately 90 mmHg. In order to simplify the patient's medical regimen and provide her with a regular rhythm at physiologically appropriate heart rates, AV junction ablation was recommended.

## Procedure

**Figure 1:**
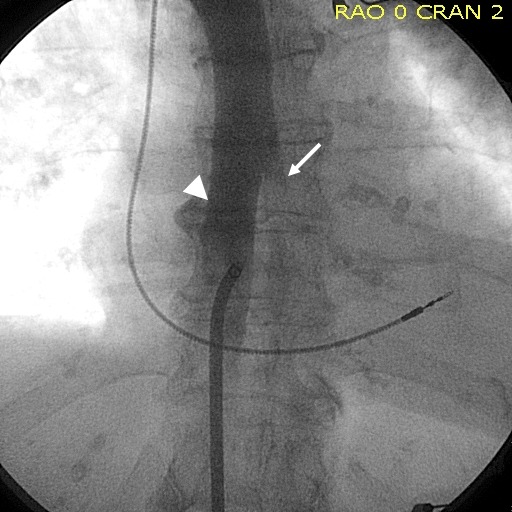
Hemiazygos vein (white arrow) merging from the left side into the middle portion of the azygos vein continuation of the interrupted IVC (white arrow head).

**Figure 2:**
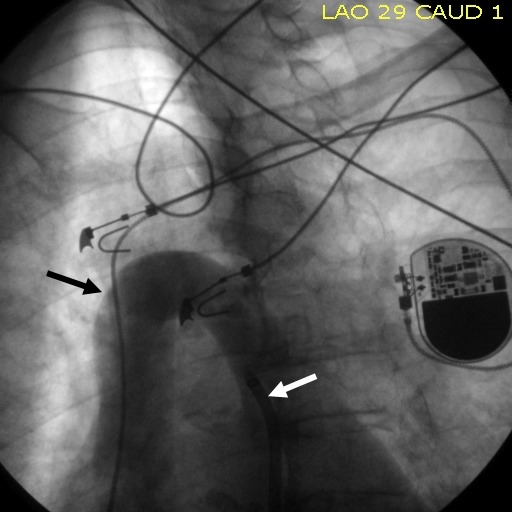
Azygos vein looping around the right hilum (black arrow) to drain into the SVC. The SRO sheath is visible in the loop (white arrow).

**Figure 3:**
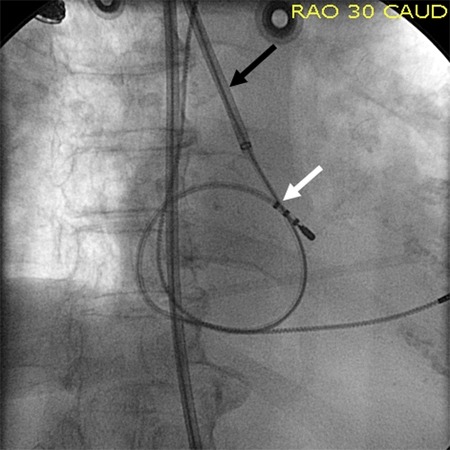
SRO sheath (black arrow) is visible in the effective arch made by the azygos vein. The ablation catheter is looped (white arrow) inside the right ventricle to simulate the inferior ablation approach.

The patient gave informed consent for the procedure and was brought to the electrophysiology laboratory. An 8 Fr venous sheath was easily inserted into the right femoral vein, through which a 7 Fr Blazer thermistor-controlled ablation catheter was passed into the central venous circulation. The catheter tip was easily advanced under fluoroscopic guidance into the cardiac silhouette, but surprisingly could not be directed into the right ventricle. Very low amplitude electrograms were also noted, suggesting that the catheter was not intra-cardiac. The ablation catheter was removed, and the 8 Fr femoral venous sheath was exchanged over a standard 0.035" J-wire for a long Schwartz right 0 (SRO) sheath. The ablation catheter was then passed through the SRO into the cardiac silhouette a second time, but again could not be advanced into the right ventricle. The catheter was removed and a series of venograms performed through the SRO sheath, each with 20 cc of nonionic contrast. The first was in the PA projection, with the tip of the sheath at the level of the mid chest (Figure 1). A second was done at the same level, but in the LAO projection. A final venogram was done with the sheath pulled back to the renal vein level of the inferior vena cava (IVC). At that point, it became evident that the IVC did not empty normally into the right atrium, but rather continued as the azygos vein, traversing through the chest behind the heart and emptying into the superior vena cava (SVC). The SRO sheath was successfully advanced around this effective arch (Figure 2), and the tip of the SRO sheath brought down to the right atrial level. The ablation catheter was then passed through the sheath and the tip taken across the tricuspid valve, both directly and subsequently looped in the right atrium to simulate an inferior approach (Figure 3). Since a stable His bundle electrogram could not be obtained in either position the ablation was done anatomically. Using the direct approach several tricuspid valve annulus positions were selected for delivery of a series of radiofrequency ablations to the suspected AV node location. Seven 60-second deliveries were made at 50 W power at 60ºC. Complete heart block was successfully attained and maintained thereafter. Subsequent to the ablation the patient's AV nodal blocker therapy was discontinued.

## Discussion

**Figure 4:**
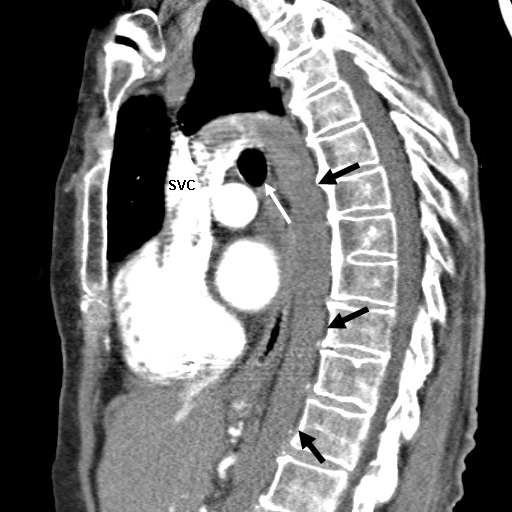
Chest CT with contrast, sagittal section, shows the azygos vein (black arrows)looping over the right bronchus (white arrow) and draining into the SVC just prior to entering the right atrium.

IVC anomalies can potentially interfere with electrophysiological studies (EPS) and catheter ablations. The incidence of systemic venous anomalies in patients undergoing cardiac catheterization for congenital heart disease ranges between 0.2 % and 1.3 % [1]. The most frequently seen are persistence of the left SVC, with or without a right SVC, and azygos/hemiazygos vein continuation of an interrupted IVC [1]. These anomalies may be isolated or associated with other anomalies [1]. Following our patient's procedure, review of a previous abdominal CT scan revealed levocardia with abdominal situs inversus. The stomach and pancreas were on the right side, and the liver was displaced to the left extending across the anterior abdomen. Multiple splenules were located in the right upper quadrant with absence of a normal spleen. There was interruption of the IVC which continued as a dilated azygos vein (Figure 4). Even though reported in the previous CT scan, unfortunately it was not noted in the echocardiograms and during the pacemaker placement earlier. This picture of anatomical abnormality is consistent with left atrial appendage isomerism (LAI), a type of heterotaxic syndrome.

Heterotaxic syndromes [2] are characterized by failure of right-to-left differentiation during embryologic development, which leads to situs inversus and anomalies of the systemic and pulmonary venous return. It is further categorized into two subtypes, left and right atrial appendage isomerism (RAI). In patients with LAI the infra-hepatic portion of the inferior vena cava is frequently absent and the venous return from the lower part of the body enters the superior vena cava via the azygos vein. In patients with RAI, the right and left hepatic veins may enter the ipsilateral sides of the common atrium, remaining separate from the inferior vena caval entrance. Polysplenia, ventricular septal defects and pulmonic stenosis are common in LAI. Neither of the latter two was present in our patient. Importantly, atrial arrhythmias occur frequently in LAI and likely explain our patient's longstanding history of palpitations [3]. Atrial pacing is known to decrease arrhythmia recurrence in adult patients with LAI [3].

There have been numerous case reports describing ablations in patients with venous anomalies and tachyarrhythmias, mostly employing the superior approach [4] and also few using the inferior approach [5]. In the current paper we report the successful ablation of the AV junction using the inferior approach in a case of interrupted IVC with azygos vein continuation draining into the SVC. The SRO sheath may be more effective than a simple short venous sheath in this situation.

Potential alternative venous access approaches in this patient would have been via the left internal jugular, right internal jugular, or right subclavian veins. Since the left subclavian vein had been used for prior pacemaker lead placement, this approach would not have been suitable. In each of these approaches, the ablation catheter enters the right heart from the superior vena cava, similar to the catheter entry from the azygos continuation of the inferior vena cava as in this case. Had our procedure been unsuccessful, one of these alternative approaches would have been appropriate. Alternatively, Stereotaxis Robotic Navigation equipment to direct the ablation catheter tip to the distal AV nodal area could have been employed.

## Conclusion

In patients with the syndrome of interrupted IVC and azygos vein continuation, AV junction ablation may be successfully performed using the inferior approach.
